# Validation of a registry-derived risk algorithm based on treatment protocol as a proxy for disease risk in childhood acute lymphoblastic leukemia

**DOI:** 10.1186/1471-2288-13-68

**Published:** 2013-05-30

**Authors:** Sumit Gupta, Jason D Pole, Astrid Guttmann, Lillian Sung

**Affiliations:** 1 Division of Haematology/Oncology and Program in Child Health Evaluative Sciences, Hospital for Sick Children, 555 University Avenue, Toronto, Ontario, M5G 1X8, Canada; 2 Pediatric Oncology Group of Ontario, 480 University Avenue, Suite 1014, Toronto, Ontario, M5G 1V2, Canada; 3 Institute for Clinical Evaluative Sciences, G1 06, 2075 Bayview Avenue, Toronto, Ontario, M4N 3M5, Canada; 4 Division of Paediatric Medicine, Hospital for Sick Children, 555 University Avenue, Toronto, Ontario, M5G 1X8, Canada; 5 Department of Paediatrics and Institute for Health, Policy Management and Evaluation, University of Toronto, 155 College Street, Suite 425, Toronto, Ontario, M5T 3M6, Canada

**Keywords:** Acute lymphoblastic leukemia, Administrative data, Health services research, Registries, Validation

## Abstract

**Background:**

Administrative databases and cancer registries are frequently used to conduct population-based research, but often lack clinical data necessary for risk stratification. Our objective was to determine the criterion validity of a risk-stratification algorithm based on treatment characteristics available from a pediatric cancer registry as a proxy for disease risk, by comparing it to traditional biology-based risk classifications.

**Methods:**

We identified all children with acute lymphoblastic leukemia diagnosed at a single institution between January 2000 and June 2011, and linked them to a population-based cancer registry. Several risk algorithms were then constructed using disease risk variables collected through chart review by a pediatric oncologist, and compared to a risk algorithm based on treatment protocol name and age, available from the registry.

**Results:**

Of 596 patients identified, 579 (97.1%) met inclusion criteria and were successfully linked. The registry-based algorithm showed almost perfect agreement with a biology-based algorithm based on age, initial white blood cell count, immunophenotype and cytogenetics (kappa=0.85, 95^th^ confidence interval 0.81-0.90). Discrepant cases were often due to the presence of unusual high risk features not captured by standard disease-risk variables but reflected in clinicians’ choices of higher intensity treatment protocols.

**Conclusions:**

Protocol name represents a valid proxy of disease risk, allowing for risk stratification while conducting comparative effectiveness research using cancer registries and health services data. Future studies should examine the validity of treatment-based risk algorithms in other malignancies and using other treatment characteristics commonly found in health services data, such as the receipt of specific chemotherapeutic agents.

## Background

The past several decades have witnessed remarkable advances in the treatment of childhood cancer, with overall cure rates now exceeding 80% [1-3]. However, in many cases significant differences exist between outcomes reported from clinical trials and population-based data. For example, Hunger et al. recently reported that children over the age of 15 years with acute lymphoblastic leukemia (ALL) treated on Children’s Oncology Group clinical trials between 2000–2005 had a 5-year survival rate of 76% [4]. By contrast, registry data for 15–19 year olds diagnosed with ALL over a similar time period showed a far lower 5-year survival of 50.1% [3]. In addition to allowing better capture of population survival trends, cancer registries and health services databases have also been used in pediatric oncology to conduct comparative effectiveness research, identify survivors at high risk of long term medical and socioeconomic adverse effects, and monitor the uptake of new therapeutic interventions [5-9].

While routinely collected population-based data holds significant potential, it can also introduce new biases. One major limitation in many of these datasets is the inability to risk stratify patients. In childhood cancer, detailed staging, histologic, genetic and response-based information is used to determine the risk of mortality and other adverse outcomes; prognosis and treatment can vary widely within a single malignancy based on this information [10,11]. These detailed biologic data are rarely collected by cancer registries or administrative databases, as highlighted by a recent review of data sources for cancer comparative effectiveness research [12]. Thus important potential confounding information is often unavailable, limiting confidence in the conclusions of studies using these resources.

A valid method of risk stratification using information available in population-based databases would increase the contribution of these data. Treatment-based risk assignment may offer such a method. In pediatric cancer, treatment intensity is often based on disease risk and biologic prognostic factors; high-risk subtypes of a particular malignancy will receive higher intensity treatment [10,11,13]. Treatment information is often collected in population-based databases: cancer registries may collect the names of treatment protocols while health services databases may collect information on the administration of particular chemotherapeutic agents [8,14].

Our objective was therefore to determine the criterion validity of a registry-based risk-stratification algorithm using treatment protocol name and age by comparing it to several traditional biology-based risk classifications. We undertook this in a single-institution cohort of children with ALL.

## Methods

### Study population

The study population included all children diagnosed with primary ALL between June 1, 2000 and December 31, 2011 at The Hospital for Sick Children, Toronto, Canada. The Hospital for Sick Children is a pediatric tertiary care institution that sees over 300 new cases of childhood cancer per year. Non-Ontario residents, children for whom no active treatment was pursued, and children transferred to other centers within the first month of treatment were excluded. Patients were identified using a local institutional electronic database. ALL was chosen as it has one of the most refined risk determination classifications in pediatric oncology, incorporating multiple biologic factors [10].

### Data sources and variables

Factors collected for each patient by chart review were: age at diagnosis, initial white blood cells (WBC) at diagnosis, immunophenotype/lineage, leukemia cytogenetics, and the presence of minimally residual disease (MRD) at the end of induction therapy. Cytogenetic abnormalities considered high risk included t(9;22) (BCR-ABL), hypodiploidy (<45 chromosomes), and any 11q23 (MLL) rearrangement [15,16]. MRD was assessed by flow cytometry; ≥0.01% residual blasts in bone marrow at the end of induction was considered positive [17]. These variables were chosen *a priori* based on both their accepted use in contemporary ALL risk stratification and the ease of their availability in patient charts [10].

POGONIS is a population-based registry that prospectively captures all cases of pediatric cancer diagnosed and treated at one of the five tertiary pediatric oncology centers in Ontario. POGONIS personnel assign each patient a unique numeric identifier, which is retained both by the treating centers and POGONIS. This number was therefore used to link study patients to POGONIS. Approximately 98% of Ontario children with cancer aged 0–14, as identified by the Ontario Cancer Registry (OCR), are captured in POGONIS [14]. Basic demographic, treatment and outcome variables are available in POGONIS. Though various biologic fields have been introduced into POGONIS at different times, they are variably collected between centers and often incompletely available. Therefore only treatment protocol and age at diagnosis were utilized from POGONIS for this study.

Multiple protocols for an individual child could be listed in POGONIS if clinicians changed treatment based on toxicity, non-response, or new prognostic information. Any subsequent protocol recorded within four weeks of the start of the first protocol was therefore identified and compared to the first. In such cases, the more specific protocol was ultimately assigned to the patient. For example, the specific protocol “AALL0331” was chosen over the more generic “three drug induction”. Where two specific protocols were identified, the one with a later start date was chosen in order to better reflect risk prognosticators available after diagnosis.

A pediatric oncologist identified patients, determined eligibility and conducted the chart abstraction for all patients. At the time of chart review, the abstractor was blinded to all patient registry data.

### Creation of risk algorithms

Biology-based risk algorithms were created using variables abstracted by chart review while a registry-based risk algorithm was created using treatment protocol name listed in POGONIS and age at diagnosis. Multiple biology-based risk algorithms were constructed using these data to divide patients into standard and high risk strata. Within each of these algorithms, the presence of any high risk feature resulted in classification in the high risk stratum. The first algorithm utilized only age and presenting WBC according to the National Cancer Institution/Rome criteria (standard risk = age ≥1 year and <10 years, and WBC <50×10^9^/L) [18]. Subsequent algorithms added additional prognosticators in order to produce sequentially more sophisticated risk classifications using the following high risk classifiers: T cell immunophenotype, poor risk cytogenetics, and presence of MRD (Table 1). Where a prognosticator was not available for a particular patient, the algorithm treated it as non-informative.

**Table 1:** Biology-based risk algorithms using data available from chart review

	**Age (years)**		**WBC (x10**^**9**^**/L)**		**Immunophenotype**		**Cytogenetics**^**&**^		**MRD**^**#**^	
	**1 - 9**	**<1, ≥10**	**<50**	**≥50**	**B**	**T**	**Low risk**	**High risk**	**Negative**	**Positive**
Algorithm 1*	SR	HR	SR	HR	-	-	-	-	-	-
Algorithm 2*	SR	HR	SR	HR	SR	HR	-	-	-	-
Algorithm 3*	SR	HR	SR	HR	SR	HR	SR	HR	-	-
Algorithm 4*	SR	HR	SR	HR	SR	HR	SR	HR	SR	HR

*HR* High risk, *MRD* Minimally residual disease, *SR* Standard risk, *WBC* White blood cell.

*For each algorithm, patients were classified as standard risk only in the absence of any high risk feature.

^&^High risk cytogenetics included t(9;22) (BCR-ABL), hypodiploidy (<45 chromosomes), or any 11q23 (MLL) rearrangement.

^#^MRD positivity was defined as ≥0.01 residual blasts.

All treatment protocols used during the study period were *a priori* classified as standard or high risk based on protocol inclusion and exclusion criteria (Table 2). Protocol inclusion criteria tend to mirror contemporaneous knowledge of favorable and unfavorable risk factors. Thus the inclusion criteria of past protocols do not perfectly align with modern definitions of standard or high risk. All protocols were therefore classified according to whether treating physicians at the time would have considered them standard or high risk. All protocol risk assignments were made through the consensus of two pediatric oncologists.

**Table 2:** Registry-based algorithm based on treatment protocol name and details of each treatment protocol

**Protocol**	**Lineage**	**Age (years)**	**WBC (x10**^**9**^**/L)**	**Genetics**	**Other**	**Risk assignment**
AALL02P2	T	≥10 (or)*	≥50 (or)			HR
AALL0031				t(9;22) or hypodiploidy or MLL with slow response (or)	Induction failure (or)	HR
AALL0232	B	≥10 (or)	≥50 (or)		Steroid pre treatment (or)	HR
AALL0331	B	1-9	<50			SR
AALL0434	T	>1				HR
AALL0622		>1		t(9;22)		HR
AALL0631		<1				HR
AALL0932	B	1-9	<50	No t(9;22), MLL, iAMP21, hypodiploidy		SR
CCG1991	B	1-9	<50			SR
POG9201	B	1-9	<50	Trisomy 4,10, DI>1.16 or t(12;21); No t(9;22), 1;19, MLL		SR
POG9407		<1				HR
POG9605	B	1-9	<50	No trisomy 4,10, DI>1.16, MLL, t(1;19), t(9;22)		SR
	B	≥10	<50	Trisomy 4,10 or DI>1.16; No MLL, t(1;19), t(9;22)		SR
	B	>1	≥50	Trisomy 4,10 or DI>1.16; No MLL, t(1;19), t(9;22)		SR
POG9904	B	1-9	<50	Trisomy 4,10, DI>1.16 or t(12;21); No MLL, t(1;19), t(9;22)		SR
POG9905					Neither 9904, 9906 nor AALL0031	SR
POG9906	B	M>12; F>16 (or)	>100 (or)	MLL (or)		HR
	B	Sliding scale of WBC criteria for M age 8–11 and F age 12-15				HR
Protocol C		<1 or >10 (or)	>20 (or)	t(9;22) or MLL (or)	L2 morphology, mediastinal mass, or massive LN/HSM	HR

*DI* DNA index, *F* Female, *HR* High risk, *HSM* Hepatosplenomegaly, *iAMP* Intra-amplification, *LN* – Lymphadenopathy, *M* Male, *SR* Standard risk, *WBC* White blood cell.

*(or) indicates factors for which any one was sufficient for inclusion into the protocol.

Age and protocol risk classification were then used to create a registry-based risk algorithm. Patients aged 1–9 years at diagnosis and treated on a protocol classified as standard risk were designated as standard risk. Patients <1 year at diagnosis, ≥10 years at diagnosis or treated on a protocol classified as high risk were designated as high risk.

All biology-based and registry-based risk algorithms were created prior to the collection of any patient data.

### Analysis

The agreement between the registry-based risk algorithm and each of the biology-based risk algorithms was assessed using the kappa statistic (0.00-0.20 slight; 0.21-0.40 fair; 0.41-0.60 moderate; 0.61-0.80 substantial; 0.81-1.00 almost perfect) [19]. The charts of discrepant cases were reviewed in detail in order to identify possible reasons for discordance. Statistical analyses were performed using SAS-PC software (version 9.2; SAS Institute, Cary, NC). Ethics approval was obtained from The Hospital for Sick Children.

## Results

A total of 596 patients were diagnosed during the study period. Twelve children were excluded: seven were not Ontario residents, three were transferred to other centers within a week of starting treatment, one represented a second malignancy, and one had no treatment pursued due to underlying comorbidities. Of the remaining 584, 579 (99.1%) were successfully linked to POGONIS. All five unlinked patients were diagnosed in the last 21 days of 2011 and had therefore not yet been registered in POGONIS at the time of data analysis.

The median age of the cohort was 4 years (interquartile range 3–8). A total of 343 (59%) patients were male. Disease-related characteristics of the cohort can be seen in Table 3. MRD results were only available on approximately half of the cohort as this investigation was only introduced into routine practice in 2005. The vast majority of patients were treated with defined protocols; treatment protocols were assigned for all but four of the study patients (575/579; 99.3%). Two patients died prior to or shortly after starting treatment, while no reason could be identified for the lack of listed protocol in the remaining two. The most commonly used protocols were from the Children’s Oncology Group: AALL0331 and AALL0232, accounting for 184 (31.8%) and 107 (18.48%) of patients respectively.

**Table 3:** Disease-related characteristics of overall study cohort and distribution of each characteristic by biology-based algorithms (N=579)

		Algorithm 1*		Algorithm 2*		Algorithm 3*		Algorithm 4*	
		**Age, WBC**		**Algorithm 1+ immunophenotype**		**Algorithm 2+cytogenetics**		**Algorithm 3+MRD**	
	**Overall**	**SR**	**HR**	**SR**	**HR**	**SR**	**HR**	**SR**	**HR**
Overall	579 (100)	359 (62)	220 (38)	347 (60)	232 (40)	340 (59)	239 (41)	324 (56)	255 (44)
Immunophenotype									
B	522 (90)	347 (66)	175 (34)	347 (66)	175 (34)	340 (65)	182 (35)	324 (62)	198 (10)
T	57 (10)	12 (21)	45 (79)	0 (0)	57 (100)	0 (0)	57 (100)	0 (0)	57 (100)
Cytogenetics									
High risk	36 (6)	7 (19)	29 (81)	7 (19)	29 (81)	0 (0)	36 (100)	0 (0)	36 (100)
MLL rearrangement	19 (3)	3 (16)	16 (84)	3 (16)	16 (84)	0 (0)	19 (100)	0 (0)	19 (100)
t(9;22) (BCR-ABL)	10 (2)	2 (20)	8 (80)	2 (20)	8 (80)	0 (0)	10 (100)	0 (0)	10 (100)
Hypodiploidy	7 (1)	2 (29)	5 (71)	2 (29)	5 (71)	0 (0)	7 (100)	0 (0)	7 (100)
Standard risk	542 (94)	352 (65)	190 (35)	340 (63)	202 (37)	340 (63)	202 (37)	324 (60)	218 (40)
Hyperdiploidy	183 (32)	147 (80)	36 (20)	147 (80)	36 (20)	147 (80)	36 (20)	143 (78)	40 (22)
t(12;21) (TEL-AML)	144 (25)	116 (81)	28 (19)	116 (81)	28 (19)	116 (81)	28 (19)	115 (80)	29 (20)
t(1;19) (E2A-PBX)	22 (4)	12 (55)	10 (45)	12 (55)	10 (45)	12 (55)	10 (45)	10 (45)	12 (55)
No specific lesion	193 (33)	77 (40)	116 (60)	65 (34)	128 (66)	65 (34)	128 (66)	56 (29)	137 (71)
Missing	1 (0)	0 (0)	1 (100)	0 (0)	1 (100)	0 (0)	1 (100)	0 (0)	1 (100)
MRD									
Negative	258 (45)	189 (73)	69 (27)	189 (73)	69 (27)	185 (72)	73 (28)	185 (72)	73 (28)
Positive	38 (5)	18 (47)	20 (53)	18 (47)	20 (53)	16 (42)	22 (58)	0 (0)	38 (100)
Died prior to test	6 (1)	1 (17)	5 (83)	1 (17)	5 (83)	1 (17)	5 (83)	1 (17)	5 (83)
Not performed	277 (48)	151 (55)	126 (45)	139 (50)	138 (50)	139 (50)	138 (50)	139 (50)	138 (50)

All values represent N (%).

*IQ* , Interquartile range; *HR*, High risk; *MRD*, Minimally residual disease; *N*, Number; *SR*, Standard risk; *WBC*, White blood cell.

The number of children classified as standard risk by the biology-based algorithms varied from 62.0% (algorithm 1 [simplest] - age and WBC) to 56.0% (algorithm 4 [most complex] – incorporating all factors of age, WBC, immunophenotype, cytogenetics and MRD) (Table 3). The registry-based algorithm classified 56.7% of patients as standard risk. Table 3 also illustrates the distribution of each disease characteristic by risk category, and its variation across algorithms.

Table 4 shows the agreement between the registry-based classification and the biology-based algorithms. Agreement was excellent (*k* ≥ 0.80) in all cases [19,20]. The best agreement as judged by the kappa statistic was between the registry-based algorithm and algorithms 2 (age, WBC, and immunophenotype) and 3 (age, WBC, immunophenotype and cytogenetics).

**Table 4:** Measures of agreement between the registry-based algorithm and the biology-based algorithms*

**Biology-based algorithms**	**Sensitivity**	**Specificity**	**PPV**	**NPV**	**Kappa, 95**^**th **^**CI**
Algorithm 1 (Age, WBC)	0.95	0.88	0.83	0.97	0.80 (0.76-0.86)
Algorithm 2 (Age, WBC, immunophenotype)	0.95	0.91	0.88	0.97	0.85 (0.81-0.89)
Algorithm 3 (Age, WBC, immunophenotype, cytogenetics)	0.94	0.96	0.89	0.96	0.85 (0.81-0.90)
Algorithm 4 (Age, WBC, immunophenotype, cytogenetics, MRD)	0.90	0.93	0.91	0.92	0.83 (0.78-0.87)

*Note that sensitivity, specificity, PPV and NPV were calculated for the ability of the registry-based algorithm to correctly identify high risk patients as defined by the various biology-based algorithms.

*CI*, Confidence interval; *MRD*, Minimally residual disease; *NPV*, Negative predictive value; *PPV*, Positive predictive value; *WBC*, White blood cell.

A total of 14 patients were classified as standard risk by the registry-based algorithm but as high risk by the biology-based algorithm 3. In two cases, this was due to data entry errors within POGONIS. In the remaining 12 cases, the misclassification was due to disease features currently known to be adverse prognosticators but which were not considered so at the time of the patient’s presentation (e.g. specific high-risk cytogenetic abnormalities). A further 27 patients were classified as high risk by the registry-based algorithm and as standard risk by the biology-based algorithm 3. Seven cases were due to data entry errors, predominantly in protocol name. Twelve cases were due to the presence of risk factors other than the *a priori* specified biology-based factors obtained by chart review, such as central nervous system involvement, testicular involvement, and steroid pre-treatment. In the remaining cases, clinicians chose high-risk treatment protocols for individualized reasons despite patients meeting standard risk criteria. A common example among these remaining cases involved the WBC count at diagnosis: several children presented with counts of <50×10^9^/L (thus meeting standard risk criteria), but which then shortly rose to ≥50×10^9^/L prior to the start of therapy.

## Discussion

We found that for children with ALL, a risk stratification system based on treatment protocol as recorded in a population-based pediatric cancer registry was a valid proxy of disease risk as compared to biology-based risk algorithms. Agreement between the registry-based algorithm and the biology-based algorithm incorporating age, WBC, immunophenotype and cytogenetics was almost perfect, with a kappa of 0.85 (95% confidence interval 0.81 to 0.90).

Though still almost perfect, agreement between the registry-based algorithm was lower with algorithm 1 (age, WBC; *k* = 0.80) and with algorithm 4 (all biologic prognosticators, including MRD; *k* =0.83). The registry-based classification was likely superior to the simplest algorithm incorporating only age and initial WBC (algorithm 1), as a portion of children considered standard risk by age and WBC will in fact have high risk immunophenotypic or cytogenetic features (Table 3); this was reflected in the treatment given to them. By contrast, as MRD results are only available approximately five weeks after starting treatment, it is unlikely that our protocol-based algorithm as currently defined captured MRD-based changes in treatment. However, high risk cytogenetics and MRD positivity are often related [17,21]. Algorithms that take cytogenetics into account either directly or indirectly (i.e. through treatment protocol) will therefore reflect a portion of the prognostic ability of MRD. It should also be noted that differences between kappa statistics in this study were small, and may in fact have been due to chance alone.

Though a minority of discrepant cases was due to data entry errors, the majority could be classified into one of three categories. The first group of discordant cases was secondary to the discrepancy between current and past knowledge of disease prognosticators. In ALL for example, new cytogenetic abnormalities associated with poor outcomes continue to be discovered [22,23]. New discoveries take time to be incorporated into clinical practice, such that past children with these risk features may not have had their treatment modified. This resulted in their misclassification as standard risk by the registry-based algorithm.

In the second group, several children had high risk factors that were not specified *a priori* by the standardized chart review. For example, the inadvertent pre-treatment with steroid of children with ALL is widely considered to increase the risk for poor outcome [24]. Despite this, investigators rarely present risk stratification by steroid pretreatment when reporting outcomes, likely due to its relative rarity and inconsistent reporting [10,17,25]. Such prognosticators are also difficult to obtain, even through chart review. It is worth noting that in the current validation study, treating clinicians were of course aware of these factors and accordingly prescribed higher intensity treatment protocols. Registry-based algorithms incorporating treatment protocol therefore seem to accurately reflect the presence of such prognosticators.

Finally, in the third group, patients met technical standard risk criteria but were nonetheless treated on high risk protocols by their physicians. For example, NCI standard risk criteria are based on the first WBC at the treating institution. In several cases however, when a presenting WBC <50×10^9^/L rapidly increased to ≥50×10^9^/L prior to treatment initiation, clinicians chose to base risk stratification on the latter value. Again, treatment protocol functioned as a proxy of physicians’ overall impression of disease risk, both when physicians adhered to or deviated from standardized risk criteria. These examples also illustrate how the utility of cancer registries for population-based research is dependent on the alignment of data capture strategies with clinical applicability [12].

Strengths of this study include the large sample size and the ability to successfully link local data to population-based registry records. A significant limitation is the ability to generalize our findings to different malignancies and treatment protocols. Some cooperative groups treat ALL with protocols whose names do not differ by risk strata, or where multiple treatment strata are contained within a single protocol name. Osteosarcoma provides another example; though the presence of metastases carries a far worse survival than localized disease, the treatment is generally the same [26]. Treatment-based risk algorithms would therefore not be a valid proxy of disease risk. In addition, some population-based registries may not collect treatment protocol names. Other ways of defining treatment intensity such as the use of specific chemotherapeutic agents or length of treatment have been used but require validation [27]. Even where other registries do collect protocol name, local validation analyses similar to that carried out in this study are likely to still be necessary before further analyses requiring risk stratification can be conducted.

A second limitation concerns the generalizability to other jurisdictions. It is possible that treatment assignment practices vary between institutions, with other centers less likely to use standardized chemotherapy protocols. However, pediatric oncology enjoys a greater degree of standardization than most medical disciplines. Almost all centers treating childhood cancer belong to large cooperative trial groups, and previous research has shown that a majority of cancer patients less that 15 years of age are registered in clinical trials across virtually all metropolitan and rural areas across the United States [28,29]. Finally, while the method presented in this paper is a valid way of approximating disease risk, and therefore in predicting survival and relapse-based outcomes, its ability to predict other outcomes (e.g. long-term side effects of treatment) is unknown.

## Conclusions

In conclusion, in this study we found that a registry-derived algorithm based on treatment protocol name and age at diagnosis was a valid method of approximating disease risk. Future studies may consider using treatment-based algorithms for the purpose of risk stratification when conducting registry-based research in pediatric oncology, though validation in other malignancies and using other indicators of treatment intensity is required.

## Abbreviations

ALL: Acute lymphoblastic leukemia; MRD: Minimal residual disease; OCR: Ontario cancer registry; POGONIS: Pediatric oncology group of ontario networked information system; WBC: White blood cell.

## Competing interests

The authors declared that they have no competing interest.

## Authors’ contributions

SG conducted the chart review and performed the analyses. All authors participated in the design of the study and helped draft the manuscript. All authors read and approved the final manuscript.

## Pre-publication history

The pre-publication history for this paper can be accessed here:

http://www.biomedcentral.com/1471-2288/13/68/prepub
